# Tetra­kis[(3-hydroxy­prop­yl)dimethyl­ammonium] tetra-μ-acetato-κ^8^ *O*:*O*′-bis­[chloridocuprate(II)](*Cu—Cu*) dichloride

**DOI:** 10.1107/S160053680900662X

**Published:** 2009-02-28

**Authors:** Muhammad Shahid, Muhammad Mazhar, Matthias Zeller, Allen D. Hunter

**Affiliations:** a Department of Chemistry, Quaid-i-Azam University, Islamabad 45320, Pakistan; b STaRBURSTT-Cyberdiffraction Consortium at YSU, and Department of Chemistry, Youngstown State University, 1 University Plaza, Youngstown, Ohio 44555-3663, USA

## Abstract

The title compound (C_5_H_14_NO)_4_[Cu_2_(CH_3_COO)_4_Cl_2_]Cl_2_, consists of a pair of Cu^II^ ions bridged by four acetate groups, resulting in a Cu_2_(CH_3_COO)_4_ unit, four (3-hydroxy­prop­yl)dimethyl­ammonium cations (two crystallographically independent pairs) and two chloride anions. The Cu atoms at both termini are bonded to chloride anions. The latter are hydrogen bonded to one of the two pairs of crystallographically independent (3-hydroxy­prop­yl)dimethyl­ammonium cations. The Cu_2_(CH_3_COO)_4_ unit is located on a crystallographic inversion center, and the geometry around each metal center is close to octa­hedral. The Cl—Cu—Cu angles are nearly linear [177.48 (2)°] and the Cu—O bond lengths are in the range 1.9712 (18)–1.9809 (19) Å. The Cu⋯Cu separation between the two acetate-bridged Cu^II^ centers is 2.6793 (8) Å. The packing of the crystal structure is dominated by N—H⋯Cl hydrogen bonding between the ammonium groups and the chloride anions, as well as by O—H⋯O and O—H⋯Cl hydrogen bonds. One of the 3-hydroxypropyldimethylammonium cations shows orientational disorder with an occupancy ratio of 0.812 (4): 0.188 (4).

## Related literature

For the structure of binuclear copper(II) complexes, see: Ackermann *et al.* (2000 ▶); Shahid, Mazhar, Helliwell *et al.* (2008 ▶). For reports on the X-ray diffraction analysis of cupric acetate hydrate, Cu_2_(CH_3_COO)_4_(H_2_O)_2_, see: Van Niekerk & Schoening (1953 ▶); de Meester *et al.* (1973 ▶); Nieger (2001 ▶); Ferguson & Glidewell (2003 ▶); Steed *et al.* (1998 ▶); Vives *et al.* (2003 ▶); Golzar Hossain (2007 ▶); Mahmoudkhani & Langer (1998 ▶). For the neutron-diffraction analysis of the same compound, see: Brown & Chidambaram (1973 ▶). For details concerning the geometric parameters of organo-copper complexes, see: Shahid, Mazhar, Malik *et al.* (2008 ▶); Shahid *et al.* (2009 ▶); Zhang *et al.* (2004 ▶).
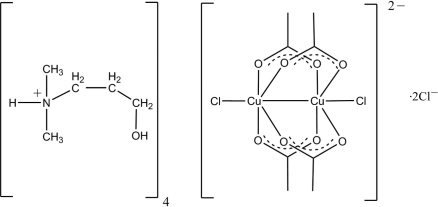


## Experimental

### 

#### Crystal data

(C_5_H_14_NO)_4_[Cu_2_(C_2_H_3_O_2_)_4_Cl_2_]Cl_2_
*M* *_r_* = 921.74
Monoclinic, 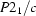

*a* = 11.438 (3) Å
*b* = 11.266 (3) Å
*c* = 16.876 (4) Å
β = 97.940 (5)°
*V* = 2153.8 (9) Å^3^
*Z* = 2
Mo *K*α radiation
μ = 1.29 mm^−1^
*T* = 100 K
0.39 × 0.33 × 0.30 mm

#### Data collection

Bruker SMART APEX CCD diffractometer
Absorption correction: multi-scan (TWINABS; Sheldrick, 2007 ▶) *T*_min_ = 0.562, *T*_max_ = 0.679
19624 measured reflections
5259 independent reflections
4776 reflections with *I* > 2σ(*I*)
*R*_int_ = 0.030

#### Refinement

*R*[*F* ^2^ > 2σ(*F* ^2^)] = 0.036
*wR*(*F* ^2^) = 0.094
*S* = 1.02
5259 reflections
246 parameters
1 restraint
H atoms treated by a mixture of independent and constrained refinement
Δρ_max_ = 1.67 e Å^−3^
Δρ_min_ = −0.66 e Å^−3^

### 

Data collection: *APEX2* (Bruker, 2008 ▶); cell refinement: *SAINT* (Bruker, 2008 ▶); data reduction: *SAINT* and *CELL* NOW (Sheldrick, 2005 ▶); program(s) used to solve structure: *SHELXTL* (Sheldrick, 2008 ▶); program(s) used to refine structure: *SHELXTL*; molecular graphics: *SHELXTL*; software used to prepare material for publication: *SHELXTL*.

## Supplementary Material

Crystal structure: contains datablocks I, global. DOI: 10.1107/S160053680900662X/su2095sup1.cif

Structure factors: contains datablocks I. DOI: 10.1107/S160053680900662X/su2095Isup2.hkl

Additional supplementary materials:  crystallographic information; 3D view; checkCIF report

## Figures and Tables

**Table 1:** Hydrogen-bond geometry (Å, °)

*D*—H⋯*A*	*D*—H	H⋯*A*	*D*⋯*A*	*D*—H⋯*A*
N1—H1⋯Cl2	0.93	2.15	3.072 (3)	169
N1*B*—H1*B*⋯Cl1^i^	0.93	2.53	3.404 (13)	156
N2—H2⋯Cl1	0.93	2.16	3.074 (2)	166
O5—H5⋯O6^ii^	0.837 (19)	1.99 (2)	2.810 (3)	166 (4)
O6—H6⋯Cl2^iii^	0.84	2.25	3.082 (2)	173

Symmetry codes: (i) 
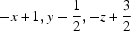
; (ii) 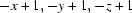; (iii) 
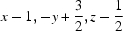
.

**Fig. 1.**
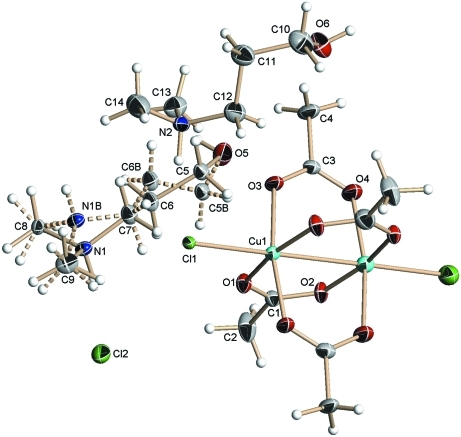
Molecular structure of the title compound showing the atom-labelling scheme and displacement ellipsoids (50% probability level). Minor moiety atoms of the disordered (dimethylammonium)propanol cation have been omitted for clarity.

**Fig. 2.**
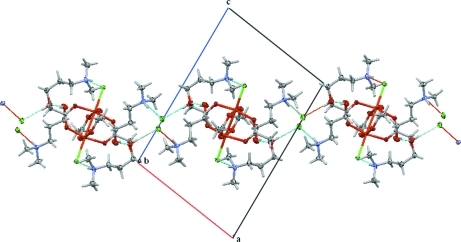
View along the a axis of the title compound, showing one of the H-bonded planes. H-bonding interactions are indicated by dashed blue lines.

**Fig. 3.**
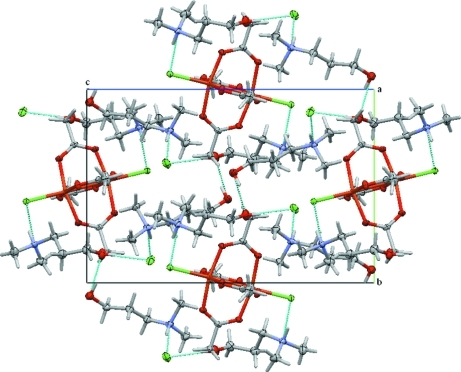
View of the title compound down the b axis (perpendicular to one of the hydrogen bonded planes). H-bonding interactions are indicated by dashed blue lines.

## supplementary crystallographic information

### Comment

In relation to our previous work on the structural chemistry of copper complexes (Shahid, Mazhar, Helliwell *et al.*, 2008) we described here the crystal structure of the title compound. It consists of a centrosymmetric acetate bridged Cu_2_(CH_3_COO)_4_ moiety with chloride anions at both termini, four (dimethylammonium)propanol cations and two chloride anions.

In the title compound (Fig. 1) the two metal centers are related by a crystallographic inversion cernter; each has a coordination environment close to octahedral, with a CuO_4_CuCl set of ligating atoms, composed of four oxygen atoms of four bridging acetate groups, a terminal chloride atom and the second copper atom. The equatorial plane is made up of atoms O1, O2, O3 and O4 of the four bridging acetate ligands connecting both copper atoms, while the chloride ion Cl1 links to Cu1 in the axial position of the octahedron. The inversion related copper atoms are linked by a Cu—Cu bond thus completing the octahedral coordination of each copper center. The *trans* angles in the equatorial plane deviate slightly from the ideal value of 180°, and the Cu—O bond lengths fall in the range of 1.9712 (18)–1.9809 (19) Å. These values are in good agreement with the values reported for Cu_2_(CH_3_COO)_4_(H_2_O)_2_ (Van Niekerk & Schoening,1953), and in more accurate structure determinations of this compound (Meester *et al.*, 1973; Nieger, 2001; Ferguson & Glidewell, 2003; Steed *et al.*, 1998; Vives *et al.*, 2003; Golzar Hossain, 2007; Mahmoudkhani & Langer, 1998) and for similar complexes (Shahid, Mazhar, Malik *et al.*, 2008; Shahid *et al.*, 2009; Zhang *et al.*, 2004). They indicate a slightly distorted octahedral geometry around both copper centers in the complex. The length of the central Cu—Cu bond of 2.6793 (8) Å is significantly longer than the value reported for dinuclear copper (II) acetate monohydrate by X-ray diffraction (see above) as well as by neutron diffraction analysis (Brown & Chidambaram, 1973), but it does agree well with that of the only other structurally determined tetra-*µ*-acetato-*k^8^O:O'*-dicuprate(II) with two terminal chloride ligands, which was reported by Ackermann *et al.* (2000) as 2.687 Å.

In the crystal structure the terminal chlorides are hydrogen bonded to one of crystallography independent (dimethylammonium)propanol cations (Fig. 2 and Table 1). The other crystallographically indepenent dimethyl(3-hydroxypropyl) ammonium ion is disordered over two positions, with both moieties being approximate mirror images of each other (see refinement section for details). This disorder results in a significantlty different hydrogen bonding environment for the two moieties. The dominant orientation exhibits an N—H···Cl hydrogen bond of *ca* 2.15 Å between H1 and Cl2. The less prevalent moiety shows a much weaker bond with an N1B—H1B···Cl1^i^ bond distance of 2.53 Å (symmetry operator (i): -*x* + 1, *y* - 1/2, -*z* + 3/2). The packing of the crystal structure is dominated by hydrogen bonding between the ammonium N—H units and the chloride (Cl2) anions, as well as O—H···O and O—H···Cl hydrogen bonds (Fig. 3 and Table 1).

### Experimental

*N*,*N*-Dimethylaminopropanol (dmapH) (0.76 g, 7.43 mmol) and acetic acid (0.45 g, 7.43 mmol) were added to a stirred suspension of Cu(CH_3_COO)_2_.H_2_O (0.74 g, 3.72 mmol) and anhydrous CuCl_2_ (0.50 g, 3.72 mmol) in 30 ml tetrahydrofuran (THF). After two hours stirring, the mixture was vacuum evaporated to dryness and the solid was redissolved in a minimum amount of THF to give green block-shaped crystals at room temperature after 10 days.

### Refinement

The crystal under investigation was found to be non-merohedrally twinned. The orientation matrices for the two components were identified using the program Cell Now (Sheldrick, 2005). The twin operation was found to be a two fold rotation around the *a* axis. The two components were integrated using Saint implemented in Apex2, resulting in a total of 19624 reflections. 1995 reflections (1332 unique ones) involved component 1 only (mean I/sigma = 16.7), 1945 reflections (1310 unique ones) involved component 2 only (mean I/sigma = 5.6), and 15684 reflections (6317 unique ones) involved both components (mean I/sigma = 10.8). The exact twin matrix identified by the integration program was found to be 1.00091 0.00101 0.00457, 0.00080 - 1.00000 - 0.00090, -0.39803 0.00179 - 1.00091.

The data were corrected for absorption using Twinabs, and the structure was solved using direct methods with only the non-overlapping reflections of component 1. The structure was refined using the hklf 5 routine with all reflections of component 1 (including the overlapping ones) below a *d*-spacing threshold of 3/4, resulting in a BASF value of 0.118 (6). The *R*_int_ value given is for all reflections before the cutoff at d = 0.75 and is based on agreement between observed single and composite intensities and those calculated from refined unique intensities and twin fractions [Twinabs (Sheldrick, 2007)].

One of the 3-dimethylamine-propan-1-ol ligands shows orientational disorder with an occupancy ratio of 0.812 (4) to 0.188 (4), with both moieties being approximate mirror images of each other. Atoms N1, C5 and C6, which significantely overlap with their equivalent counterparts, were constrained to have the same ADPs as their equivalent partners in the minor moiety. No restraints were applied for non-hydrogen atoms.

The hydroxyl hydrogen atom bonded to O5 did not show any visible disorder [despite being part of the disordered dimethyl(3-hydroxypropyl)ammonium group] and was freely refined with an O—H distance restraint of 0.837 (19) Å. The C—H and N—H atoms were placed in calculated positions and treated as riding atoms: N-H = 0.93 Å, C-H = 0.98 - 0.99 Å, with *U*_iso_(H) = k × U_eq_(parent atom), where k is 1.2 for C-methylene and N-ammonium, and 1.5 for C-methyl and O-hydroxyl.

### Crystal data

**Table:**

(C_5_H_14_NO)_4_[Cu_2_(C_2_H_3_O_2_)_4_Cl_2_]Cl_2_	*F*(000) = 972
*M**_r_* = 921.74	*D*_x_ = 1.421 Mg m^−^^3^
Monoclinic, *P*2_1_/*c*	Mo *K*α radiation, λ = 0.71073 Å
Hall symbol: -P 2ybc	Cell parameters from 2075 reflections
*a* = 11.438 (3) Å	θ = 2.7–30.4°
*b* = 11.266 (3) Å	µ = 1.29 mm^−^^1^
*c* = 16.876 (4) Å	*T* = 100 K
β = 97.940 (5)°	Block, green
*V* = 2153.8 (9) Å^3^	0.39 × 0.33 × 0.30 mm
*Z* = 2	

### Data collection

**Table:**

Bruker SMART APEX CCD diffractometer	5259 independent reflections
Radiation source: fine-focus sealed tube	4776 reflections with *I* > 2σ(*I*)
graphite	*R*_int_ = 0.030
ω scans	θ_max_ = 28.3°, θ_min_ = 1.8°
Absorption correction: multi-scan (TWINABS; Sheldrick, 2007)	*h* = −15→15
*T*_min_ = 0.562, *T*_max_ = 0.679	*k* = 0→15
19624 measured reflections	*l* = 0→22

### Refinement

**Table:**

Refinement on *F*^2^	Primary atom site location: structure-invariant direct methods
Least-squares matrix: full	Secondary atom site location: difference Fourier map
*R*[*F*^2^ > 2σ(*F*^2^)] = 0.036	Hydrogen site location: inferred from neighbouring sites
*wR*(*F*^2^) = 0.094	H atoms treated by a mixture of independent and constrained refinement
*S* = 1.02	*w* = 1/[σ^2^(*F*_o_^2^) + (0.0511*P*)^2^ + 2.2181*P*] where *P* = (*F*_o_^2^ + 2*F*_c_^2^)/3
5259 reflections	(Δ/σ)_max_ = 0.001
246 parameters	Δρ_max_ = 1.67 e Å^−^^3^
1 restraint	Δρ_min_ = −0.66 e Å^−^^3^
3 constraints	

### Special details

**Table:**

Geometry. All e.s.d.'s (except the e.s.d. in the dihedral angle between two l.s. planes) are estimated using the full covariance matrix. The cell e.s.d.'s are taken into account individually in the estimation of e.s.d.'s in distances, angles and torsion angles; correlations between e.s.d.'s in cell parameters are only used when they are defined by crystal symmetry. An approximate (isotropic) treatment of cell e.s.d.'s is used for estimating e.s.d.'s involving l.s. planes.
Refinement. Refinement of *F*^2^ against ALL reflections. The weighted *R*-factor *wR* and goodness of fit *S* are based on *F*^2^, conventional *R*-factors *R* are based on *F*, with *F* set to zero for negative *F*^2^. The threshold expression of *F*^2^ > σ(*F*^2^) is used only for calculating *R*-factors(gt) *etc*. and is not relevant to the choice of reflections for refinement. *R*-factors based on *F*^2^ are statistically about twice as large as those based on *F*, and *R*- factors based on ALL data will be even larger.

### Fractional atomic coordinates and isotropic or equivalent isotropic displacement parameters (Å^2^)

**Table:**

	*x*	*y*	*z*	*U*_iso_*/*U*_eq_	Occ. (<1)
C1	0.7197 (2)	0.9685 (2)	0.54155 (15)	0.0174 (5)	
C2	0.8514 (3)	0.9625 (4)	0.5660 (2)	0.0406 (9)	
H2A	0.8854	0.9046	0.5323	0.061*	
H2B	0.8683	0.9382	0.6221	0.061*	
H2C	0.8860	1.0408	0.5593	0.061*	
C3	0.4643 (2)	0.7863 (2)	0.45644 (15)	0.0166 (5)	
C4	0.4394 (3)	0.6600 (2)	0.42948 (17)	0.0239 (5)	
H4A	0.4418	0.6541	0.3718	0.036*	
H4B	0.3610	0.6366	0.4411	0.036*	
H4C	0.4991	0.6073	0.4580	0.036*	
N1	0.7861 (2)	0.7142 (2)	0.78810 (17)	0.0189 (6)	0.812 (4)
H1	0.8449	0.7666	0.7777	0.023*	0.812 (4)
C5	0.7036 (4)	0.6247 (3)	0.5698 (2)	0.0245 (8)	0.812 (4)
H5A	0.6312	0.5809	0.5778	0.029*	0.812 (4)
H5B	0.6797	0.7055	0.5511	0.029*	0.812 (4)
C6	0.7825 (4)	0.6345 (4)	0.6499 (2)	0.0252 (8)	0.812 (4)
H6A	0.8115	0.5548	0.6677	0.030*	0.812 (4)
H6B	0.8517	0.6849	0.6440	0.030*	0.812 (4)
N1B	0.7363 (11)	0.6648 (11)	0.7942 (7)	0.0189 (6)	0.188 (4)
H1B	0.6773	0.6120	0.8038	0.023*	0.188 (4)
C6B	0.7335 (17)	0.6065 (17)	0.6489 (11)	0.0252 (8)	0.188 (4)
H6B1	0.6645	0.5527	0.6406	0.030*	0.188 (4)
H6B2	0.8028	0.5573	0.6695	0.030*	0.188 (4)
C5B	0.7530 (17)	0.6512 (16)	0.5689 (11)	0.0245 (8)	0.188 (4)
H5B1	0.6895	0.7086	0.5505	0.029*	0.188 (4)
H5B2	0.8286	0.6952	0.5752	0.029*	0.188 (4)
C7	0.7144 (3)	0.6879 (3)	0.71129 (18)	0.0278 (6)	
H7C	0.7609	0.7597	0.7025	0.033*	0.188 (4)
H7D	0.6304	0.7114	0.7001	0.033*	0.188 (4)
H7A	0.6777	0.7626	0.6892	0.033*	0.812 (4)
H7B	0.6499	0.6333	0.7206	0.033*	0.812 (4)
C8	0.8470 (2)	0.6073 (2)	0.82870 (17)	0.0235 (5)	
H8D	0.8953	0.5912	0.7862	0.035*	0.188 (4)
H8E	0.8293	0.5324	0.8541	0.035*	0.188 (4)
H8F	0.8904	0.6598	0.8688	0.035*	0.188 (4)
H8A	0.9118	0.5819	0.8001	0.035*	0.812 (4)
H8B	0.7904	0.5422	0.8295	0.035*	0.812 (4)
H8C	0.8788	0.6291	0.8838	0.035*	0.812 (4)
C9	0.7160 (3)	0.7754 (3)	0.84400 (19)	0.0300 (6)	
H9D	0.6966	0.8432	0.8082	0.045*	0.188 (4)
H9E	0.7878	0.7930	0.8809	0.045*	0.188 (4)
H9F	0.6506	0.7606	0.8746	0.045*	0.188 (4)
H9A	0.6952	0.8553	0.8238	0.045*	0.812 (4)
H9B	0.7625	0.7810	0.8973	0.045*	0.812 (4)
H9C	0.6436	0.7302	0.8474	0.045*	0.812 (4)
C10	0.0463 (3)	0.6724 (3)	0.49916 (18)	0.0293 (6)	
H10A	−0.0239	0.6212	0.4849	0.035*	
H10B	0.0207	0.7563	0.4940	0.035*	
C11	0.1022 (3)	0.6472 (3)	0.58522 (18)	0.0283 (6)	
H11A	0.0406	0.6491	0.6210	0.034*	
H11B	0.1380	0.5670	0.5883	0.034*	
C12	0.1966 (3)	0.7392 (3)	0.61261 (18)	0.0267 (6)	
H12A	0.2394	0.7589	0.5672	0.032*	
H12B	0.1578	0.8125	0.6281	0.032*	
C13	0.3698 (3)	0.6112 (3)	0.6563 (2)	0.0321 (7)	
H13A	0.4199	0.5798	0.7034	0.048*	
H13B	0.4192	0.6507	0.6212	0.048*	
H13C	0.3265	0.5459	0.6272	0.048*	
C14	0.2309 (3)	0.6569 (3)	0.75181 (19)	0.0360 (7)	
H14A	0.1747	0.7164	0.7658	0.054*	
H14B	0.2929	0.6449	0.7973	0.054*	
H14C	0.1895	0.5818	0.7386	0.054*	
Cl1	0.44011 (5)	0.92078 (5)	0.71072 (4)	0.01876 (13)	
Cl2	0.99391 (6)	0.88351 (7)	0.77784 (4)	0.03015 (16)	
Cu1	0.48200 (3)	0.97068 (2)	0.574297 (18)	0.01280 (8)	
N2	0.2843 (2)	0.6984 (2)	0.68207 (14)	0.0217 (5)	
H2	0.3285	0.7651	0.6994	0.026*	
O1	0.65475 (16)	0.94688 (18)	0.59419 (11)	0.0222 (4)	
O2	0.68435 (16)	0.99580 (19)	0.46970 (11)	0.0226 (4)	
O3	0.45992 (18)	0.80971 (15)	0.52892 (11)	0.0217 (4)	
O4	0.48787 (17)	0.85928 (15)	0.40413 (11)	0.0215 (4)	
O5	0.7561 (2)	0.5676 (2)	0.50998 (13)	0.0322 (5)	
H5	0.787 (4)	0.506 (3)	0.531 (2)	0.048*	
O6	0.13091 (19)	0.6490 (2)	0.44697 (13)	0.0319 (5)	
H6	0.0969	0.6451	0.3996	0.048*	

### Atomic displacement parameters (Å^2^)

**Table:**

	*U*^11^	*U*^22^	*U*^33^	*U*^12^	*U*^13^	*U*^23^
C1	0.0166 (11)	0.0172 (11)	0.0174 (12)	0.0003 (9)	−0.0008 (9)	−0.0002 (9)
C2	0.0172 (13)	0.074 (3)	0.0297 (16)	0.0020 (15)	0.0012 (12)	0.0113 (17)
C3	0.0147 (10)	0.0146 (10)	0.0202 (12)	−0.0002 (9)	0.0012 (9)	−0.0039 (9)
C4	0.0353 (15)	0.0133 (11)	0.0241 (13)	−0.0064 (10)	0.0076 (12)	−0.0036 (10)
N1	0.0184 (13)	0.0156 (13)	0.0238 (13)	−0.0010 (10)	0.0070 (11)	−0.0008 (11)
C5	0.024 (2)	0.0215 (17)	0.0265 (16)	0.0052 (15)	−0.0011 (17)	−0.0015 (14)
C6	0.022 (2)	0.0281 (19)	0.0245 (16)	0.0078 (16)	−0.0001 (16)	−0.0018 (14)
N1B	0.0184 (13)	0.0156 (13)	0.0238 (13)	−0.0010 (10)	0.0070 (11)	−0.0008 (11)
C6B	0.022 (2)	0.0281 (19)	0.0245 (16)	0.0078 (16)	−0.0001 (16)	−0.0018 (14)
C5B	0.024 (2)	0.0215 (17)	0.0265 (16)	0.0052 (15)	−0.0011 (17)	−0.0015 (14)
C7	0.0301 (15)	0.0265 (14)	0.0254 (15)	0.0084 (11)	−0.0015 (12)	−0.0017 (12)
C8	0.0252 (13)	0.0225 (13)	0.0223 (13)	0.0028 (11)	0.0012 (11)	0.0018 (11)
C9	0.0338 (15)	0.0266 (14)	0.0325 (16)	0.0026 (12)	0.0145 (13)	−0.0064 (12)
C10	0.0205 (13)	0.0357 (15)	0.0301 (15)	0.0026 (11)	−0.0019 (11)	−0.0038 (13)
C11	0.0247 (13)	0.0304 (14)	0.0296 (15)	−0.0043 (11)	0.0031 (11)	−0.0016 (12)
C12	0.0274 (14)	0.0220 (13)	0.0288 (15)	0.0013 (11)	−0.0033 (12)	0.0007 (11)
C13	0.0256 (14)	0.0264 (14)	0.0431 (18)	0.0033 (12)	0.0003 (13)	0.0048 (13)
C14	0.0360 (17)	0.0467 (19)	0.0249 (15)	−0.0124 (15)	0.0024 (13)	0.0013 (14)
Cl1	0.0248 (3)	0.0182 (3)	0.0133 (3)	−0.0063 (2)	0.0026 (2)	−0.0005 (2)
Cl2	0.0299 (3)	0.0342 (4)	0.0242 (3)	−0.0153 (3)	−0.0042 (3)	0.0082 (3)
Cu1	0.01441 (13)	0.01100 (13)	0.01291 (14)	−0.00040 (10)	0.00160 (11)	−0.00054 (10)
N2	0.0254 (11)	0.0159 (10)	0.0215 (11)	−0.0030 (9)	−0.0048 (9)	0.0011 (9)
O1	0.0167 (8)	0.0294 (10)	0.0202 (9)	0.0011 (7)	0.0020 (7)	0.0052 (8)
O2	0.0158 (8)	0.0337 (10)	0.0180 (9)	0.0035 (8)	0.0010 (7)	0.0050 (8)
O3	0.0331 (10)	0.0135 (8)	0.0189 (9)	−0.0045 (7)	0.0054 (8)	−0.0026 (7)
O4	0.0332 (10)	0.0129 (8)	0.0187 (9)	−0.0031 (7)	0.0043 (8)	−0.0020 (7)
O5	0.0470 (13)	0.0255 (11)	0.0234 (11)	0.0082 (10)	0.0022 (10)	−0.0015 (9)
O6	0.0281 (10)	0.0425 (13)	0.0235 (10)	0.0065 (10)	−0.0021 (8)	−0.0025 (9)

### Geometric parameters (Å, °)

**Table:**

C1—O1	1.258 (3)	C8—H8F	0.9800
C1—O2	1.262 (3)	C8—H8A	0.9804
C1—C2	1.506 (4)	C8—H8B	0.9801
C2—H2A	0.9800	C8—H8C	0.9821
C2—H2B	0.9800	C9—H9D	0.9800
C2—H2C	0.9800	C9—H9E	0.9800
C3—O3	1.259 (3)	C9—H9F	0.9800
C3—O4	1.262 (3)	C9—H9A	0.9799
C3—C4	1.509 (3)	C9—H9B	0.9824
C4—H4A	0.9800	C9—H9C	0.9805
C4—H4B	0.9800	C10—O6	1.421 (4)
C4—H4C	0.9800	C10—C11	1.531 (4)
N1—C7	1.465 (4)	C10—H10A	0.9900
N1—C9	1.489 (4)	C10—H10B	0.9900
N1—C8	1.508 (4)	C11—C12	1.522 (4)
N1—H1	0.9300	C11—H11A	0.9900
C5—O5	1.399 (4)	C11—H11B	0.9900
C5—C6	1.522 (6)	C12—N2	1.506 (4)
C5—H5A	0.9900	C12—H12A	0.9900
C5—H5B	0.9900	C12—H12B	0.9900
C6—C7	1.505 (5)	C13—N2	1.493 (4)
C6—H6A	0.9900	C13—H13A	0.9800
C6—H6B	0.9900	C13—H13B	0.9800
N1B—C7	1.412 (13)	C13—H13C	0.9800
N1B—C8	1.469 (12)	C14—N2	1.475 (4)
N1B—C9	1.538 (12)	C14—H14A	0.9800
N1B—H1B	0.9300	C14—H14B	0.9800
C6B—C7	1.435 (18)	C14—H14C	0.9800
C6B—C5B	1.49 (3)	Cl1—Cu1	2.4800 (9)
C6B—H6B1	0.9900	Cu1—O3	1.9712 (18)
C6B—H6B2	0.9900	Cu1—O4^i^	1.9714 (18)
C5B—O5	1.374 (18)	Cu1—O1	1.9762 (19)
C5B—H5B1	0.9900	Cu1—O2^i^	1.9809 (19)
C5B—H5B2	0.9900	Cu1—Cu1^i^	2.6793 (8)
C7—H7C	0.9900	N2—H2	0.9300
C7—H7D	0.9900	O2—Cu1^i^	1.9809 (19)
C7—H7A	0.9902	O4—Cu1^i^	1.9714 (18)
C7—H7B	0.9902	O5—H5	0.837 (19)
C8—H8D	0.9800	O6—H6	0.8400
C8—H8E	0.9800		
			
O1—C1—O2	125.7 (2)	H8A—C8—H8B	109.5
O1—C1—C2	117.6 (2)	N1—C8—H8C	108.5
O2—C1—C2	116.7 (2)	H8A—C8—H8C	109.4
C1—C2—H2A	109.5	H8B—C8—H8C	109.5
C1—C2—H2B	109.5	N1B—C9—H9D	109.5
H2A—C2—H2B	109.5	N1B—C9—H9E	109.5
C1—C2—H2C	109.5	H9D—C9—H9E	109.5
H2A—C2—H2C	109.5	N1B—C9—H9F	109.5
H2B—C2—H2C	109.5	H9D—C9—H9F	109.5
O3—C3—O4	125.7 (2)	H9E—C9—H9F	109.5
O3—C3—C4	117.3 (2)	N1—C9—H9A	109.4
O4—C3—C4	116.9 (2)	N1—C9—H9B	109.8
C3—C4—H4A	109.5	H9A—C9—H9B	109.5
C3—C4—H4B	109.5	N1—C9—H9C	109.2
H4A—C4—H4B	109.5	H9D—C9—H9C	108.7
C3—C4—H4C	109.5	H9A—C9—H9C	109.4
H4A—C4—H4C	109.5	H9B—C9—H9C	109.5
H4B—C4—H4C	109.5	O6—C10—C11	108.8 (2)
C7—N1—C9	111.7 (2)	O6—C10—H10A	109.9
C7—N1—C8	114.1 (2)	C11—C10—H10A	109.9
C9—N1—C8	109.8 (2)	O6—C10—H10B	109.9
C7—N1—H1	107.0	C11—C10—H10B	109.9
C9—N1—H1	107.0	H10A—C10—H10B	108.3
C8—N1—H1	107.0	C12—C11—C10	110.4 (2)
O5—C5—C6	114.4 (3)	C12—C11—H11A	109.6
O5—C5—H5A	108.6	C10—C11—H11A	109.6
C6—C5—H5A	108.6	C12—C11—H11B	109.6
O5—C5—H5B	108.6	C10—C11—H11B	109.6
C6—C5—H5B	108.6	H11A—C11—H11B	108.1
H5A—C5—H5B	107.6	N2—C12—C11	113.4 (2)
C7—C6—C5	110.0 (3)	N2—C12—H12A	108.9
C7—C6—H6A	109.7	C11—C12—H12A	108.9
C5—C6—H6A	109.7	N2—C12—H12B	108.9
C7—C6—H6B	109.7	C11—C12—H12B	108.9
C5—C6—H6B	109.7	H12A—C12—H12B	107.7
H6A—C6—H6B	108.2	N2—C13—H13A	109.5
C7—N1B—C8	119.9 (8)	N2—C13—H13B	109.5
C7—N1B—C9	111.9 (8)	H13A—C13—H13B	109.5
C8—N1B—C9	109.2 (8)	N2—C13—H13C	109.5
C7—N1B—H1B	104.8	H13A—C13—H13C	109.5
C8—N1B—H1B	104.8	H13B—C13—H13C	109.5
C9—N1B—H1B	104.8	N2—C14—H14A	109.5
C7—C6B—C5B	120.5 (15)	N2—C14—H14B	109.5
C7—C6B—H6B1	107.2	H14A—C14—H14B	109.5
C5B—C6B—H6B1	107.2	N2—C14—H14C	109.5
C7—C6B—H6B2	107.2	H14A—C14—H14C	109.5
C5B—C6B—H6B2	107.2	H14B—C14—H14C	109.5
H6B1—C6B—H6B2	106.8	O3—Cu1—O4^i^	167.29 (7)
O5—C5B—C6B	116.6 (15)	O3—Cu1—O1	90.72 (8)
O5—C5B—H5B1	108.1	O4^i^—Cu1—O1	87.44 (8)
C6B—C5B—H5B1	108.1	O3—Cu1—O2^i^	87.99 (9)
O5—C5B—H5B2	108.1	O4^i^—Cu1—O2^i^	91.05 (9)
C6B—C5B—H5B2	108.1	O1—Cu1—O2^i^	167.32 (8)
H5B1—C5B—H5B2	107.3	O3—Cu1—Cl1	96.96 (6)
N1B—C7—C6B	125.9 (9)	O4^i^—Cu1—Cl1	95.74 (6)
N1—C7—C6	114.3 (3)	O1—Cu1—Cl1	97.38 (6)
N1B—C7—H7C	105.9	O2^i^—Cu1—Cl1	95.30 (6)
C6B—C7—H7C	105.9	O3—Cu1—Cu1^i^	83.76 (6)
N1B—C7—H7D	105.9	O4^i^—Cu1—Cu1^i^	83.56 (6)
C6B—C7—H7D	105.9	O1—Cu1—Cu1^i^	85.02 (6)
H7C—C7—H7D	106.2	O2^i^—Cu1—Cu1^i^	82.30 (6)
N1—C7—H7A	108.5	Cl1—Cu1—Cu1^i^	177.48 (2)
C6—C7—H7A	108.3	C14—N2—C13	112.6 (2)
N1—C7—H7B	108.9	C14—N2—C12	114.4 (2)
C6—C7—H7B	109.2	C13—N2—C12	111.5 (2)
H7A—C7—H7B	107.6	C14—N2—H2	105.9
N1B—C8—H8D	109.5	C13—N2—H2	105.9
N1B—C8—H8E	109.5	C12—N2—H2	105.9
H8D—C8—H8E	109.5	C1—O1—Cu1	121.88 (17)
N1B—C8—H8F	109.5	C1—O2—Cu1^i^	124.78 (16)
H8D—C8—H8F	109.5	C3—O3—Cu1	123.36 (16)
H8E—C8—H8F	109.5	C3—O4—Cu1^i^	123.53 (16)
N1—C8—H8A	110.0	C5B—O5—H5	109 (3)
N1—C8—H8B	109.8	C5—O5—H5	106 (3)
H8D—C8—H8B	108.2	C10—O6—H6	109.5
			
O5—C5—C6—C7	−175.3 (3)	C7—N1B—C9—N1	−67.1 (9)
C7—C6B—C5B—O5	−172.2 (12)	C8—N1B—C9—N1	68.0 (9)
C8—N1B—C7—C6B	45.4 (17)	O6—C10—C11—C12	69.3 (3)
C9—N1B—C7—C6B	175.1 (11)	C10—C11—C12—N2	−159.0 (2)
C8—N1B—C7—N1	−66.4 (10)	C11—C12—N2—C14	−53.9 (3)
C9—N1B—C7—N1	63.3 (8)	C11—C12—N2—C13	75.3 (3)
C8—N1B—C7—C6	14.5 (14)	O2—C1—O1—Cu1	−7.1 (4)
C9—N1B—C7—C6	144.3 (5)	C2—C1—O1—Cu1	172.3 (2)
C5B—C6B—C7—N1B	−155.9 (14)	O3—Cu1—O1—C1	86.9 (2)
C5B—C6B—C7—N1	−114.2 (15)	O4^i^—Cu1—O1—C1	−80.6 (2)
C5B—C6B—C7—C6	−59.1 (19)	O2^i^—Cu1—O1—C1	2.8 (5)
C9—N1—C7—N1B	−67.2 (9)	Cl1—Cu1—O1—C1	−176.03 (19)
C8—N1—C7—N1B	57.9 (9)	Cu1^i^—Cu1—O1—C1	3.18 (19)
C9—N1—C7—C6B	−162.1 (11)	O1—C1—O2—Cu1^i^	7.4 (4)
C8—N1—C7—C6B	−37.0 (12)	C2—C1—O2—Cu1^i^	−172.0 (2)
C9—N1—C7—C6	175.5 (3)	O4—C3—O3—Cu1	3.2 (4)
C8—N1—C7—C6	−59.4 (4)	C4—C3—O3—Cu1	−176.89 (18)
C5—C6—C7—N1B	151.0 (7)	O4^i^—Cu1—O3—C3	−5.5 (5)
C5—C6—C7—C6B	49.7 (18)	O1—Cu1—O3—C3	−87.0 (2)
C5—C6—C7—N1	−173.2 (3)	O2^i^—Cu1—O3—C3	80.3 (2)
C7—N1B—C8—N1	66.6 (10)	Cl1—Cu1—O3—C3	175.4 (2)
C9—N1B—C8—N1	−64.3 (8)	Cu1^i^—Cu1—O3—C3	−2.1 (2)
C7—N1—C8—N1B	−57.1 (9)	O3—C3—O4—Cu1^i^	−2.1 (4)
C9—N1—C8—N1B	69.1 (9)	C4—C3—O4—Cu1^i^	177.95 (17)
C7—N1—C9—N1B	62.5 (9)	C6B—C5B—O5—C5	54.7 (17)
C8—N1—C9—N1B	−65.1 (9)	C6—C5—O5—C5B	−51.9 (17)

Symmetry codes: (i) −*x*+1, −*y*+2, −*z*+1.

### Hydrogen-bond geometry (Å, °)

**Table:**

*D*—H···*A*	*D*—H	H···*A*	*D*···*A*	*D*—H···*A*
N1—H1···Cl2	0.93	2.15	3.072 (3)	169
N1B—H1B···Cl1^ii^	0.93	2.53	3.404 (13)	156
N2—H2···Cl1	0.93	2.16	3.074 (2)	166
O5—H5···O6^iii^	0.84 (2)	1.99 (2)	2.810 (3)	166 (4)
O6—H6···Cl2^iv^	0.84	2.25	3.082 (2)	173

Symmetry codes: (ii) −*x*+1, *y*−1/2, −*z*+3/2; (iii) −*x*+1, −*y*+1, −*z*+1; (iv) *x*−1, −*y*+3/2, *z*−1/2.
